# Chimerism practices across Canadian transplant centres: review of current standards and variations

**DOI:** 10.3389/frtra.2026.1879340

**Published:** 2026-07-31

**Authors:** Matthew Gravina, Kylie Lepic, Brian Leber, Irwin Walker, Tobias Berg, Wilson Lam, Alejandro Garcia-Horton, Shreyash Dalmia, Daria Grafodatskaya, Darci T. Butcher, Joseph Alexander Aziz, Michael Radford

**Affiliations:** 1 Department of Medicine, McMaster University, Hamilton, ON, Canada; 2 Department of Oncology, McMaster University, Hamilton, ON, Canada; 3 Escarpment Cancer Research Institute, Hamilton Health Sciences/McMaster University, Hamilton, ON, Canada; 4 Centre for Discovery in Cancer Research, McMaster University, Hamilton, ON, Canada; 5 Department of Pathology and Molecular Medicine, McMaster University, Hamilton, ON, Canada; 6 Hamilton Regional Laboratory Medicine Program, Hamilton Health Sciences, Hamilton, ON, Canada

**Keywords:** allogeneic stem cell transplant, Canada, chimerism, hematology, next generation sequencing (NGS)

## Abstract

**Background:**

Chimerism analysis is used in hematopoietic stem cell transplantation (HSCT) to evaluate donor engraftment and assess underlying disease status. Practices vary greatly amongst Canadian transplant centres, and currently, there are no consensus guidelines for testing methodology, time points, cellular source and clinical interventions.

**Objectives:**

To review the current chimerism practice patterns for Canadian transplant centres and review current literature

**Study design:**

We surveyed members of Cell Therapy and Transplant Canada (CTTC) via a web based application regarding chimerism practices nationwide with approximately half of all transplant centres responding.

**Results:**

The majority of centres used similar chimerism laboratory methodologies, though we identified key areas of heterogeneity across transplant centres, namely in the time points for testing and the cellular lineages evaluated. Implications and management of mixed donor chimerism also varied between centres resulting in variability of interventions.

**Conclusions:**

Review of current chimerism testing literature highlighted the improving sensitivities of novel testing methods available as well as testing specific cellular lineages to evaluate ongoing donor hematopoiesis. Our survey emphasizes the importance of advocating for a national standard in chimerism testing, and subsequent management based on chimerism results, to better inform clinical practice and to aid in development of prospective research.

## 1 Introduction

Allogeneic hematopoietic stem cell transplantation (HSCT) is a potentially curative therapy for malignant and non-malignant hematologic diseases. Successful HSCT requires adequate engraftment of donor stem cells within the host hematopoietic compartment, establishment of normal immunity, and expansion of donor lymphocytes to eradicate residual disease via a “graft vs. tumor” effect. Monitoring engraftment of donor-derived immune cell lineages in the post-transplant setting through chimerism testing evaluates engraftment and can predict impending relapse (1–3). Complete donor chimerism, defined as the percentage of cells of donor origin being greater than the upper limit of detection of the testing method (typically >95%–99% donor cells based on sensitivity of technique), results in the best clinical outcomes for patients and has been demonstrated to be associated with reduced rates of relapse (1, 2, 4). Conversely, patients with mixed donor chimerism have detectable levels of both donor and recipient cell lineages, which, depending on time point tested, may reflect progressing engraftment, impending graft failure or disease relapse (5, 6). Detection of increasing mixed chimerism, especially with previously established complete chimerism, has been shown to correlate with an increased likelihood of relapse and provides a window of opportunity for early therapeutic intervention, namely reduction of immunosuppression (promoting graft-vs.-tumour effect), or use of donor lymphocyte infusion (DLI) (7, 8).

Current chimerism testing practices are heterogeneous amongst transplant centres and vary with respect to local expertise, academic resources and availability of testing (9). Donor DNA interrogation methodology, frequency of testing time points, and clinical decision-making based on chimerism results are often centre-specific; previous attempts to standardize practices have not taken hold (9). Technological advancements have resulted in multiple methodologies for chimerism investigation with increased potential to improve sensitivity, precision and throughput; however, these techniques are not ubiquitously used across transplant centres (9). Short tandem repeat polymerase chain reaction (STR-PCR) and fluorescence *in situ* hybridization (FISH, for sex mismatched transplant only) have traditionally been the most commonly used methods of chimerism analysis, though novel techniques such as quantitative PCR (qPCR), digital droplet PCR (ddPCR) and next-generation sequencing (NGS) are increasingly being adopted (10, 11). Additionally, guidelines on clinical indications for chimerism testing [i.e., myeloablative (MAC) vs. non-myeloablative (NMA) conditioning], testing time points post-transplant and lineage selection are not well-defined, with limited high-quality evidence to support practice.

Although reviews of worldwide practices of chimerism testing have been published (9, 12) Canadian-specific testing practices are yet to be established. We present survey data from transplant centres across Canada to understand chimerism practices and discuss potential areas to build consensus.

## 2 Methods

### 2.1 Study design

A web-based survey consisting of 15 questions pertaining to Canadian post-transplant chimerism practices was developed (Supplementary Appendix S1). Multiple-choice questions were focused at identifying HSCT centre information in addition to general testing methods, practices, and clinical utilization of chimerism results. Multiple responses were able to be selected for certain questions by a single centre if applicable. A descriptive option was provided in select questions to give respondents the opportunity to answer, should their centre response differ from pre-selected options. The survey was piloted by adult HSCT physicians excluding the program director at Juravinski Cancer Centre, Hamilton, Ontario, Canada. This was subsequently distributed by email Nov 2025 to the 16 allogeneic transplant program directors across Canada. The survey was closed Dec 2025. A description of the survey being for research purposes was provided to respondents with consent to proceed being obtained prior to collecting responses. Institutional research ethics boards (IREB) approval was obtained to collect survey data for research purposes. Data was collected and summarized using REDcap.

## 3 Results

### 3.1 Participants

**Figure 1:**
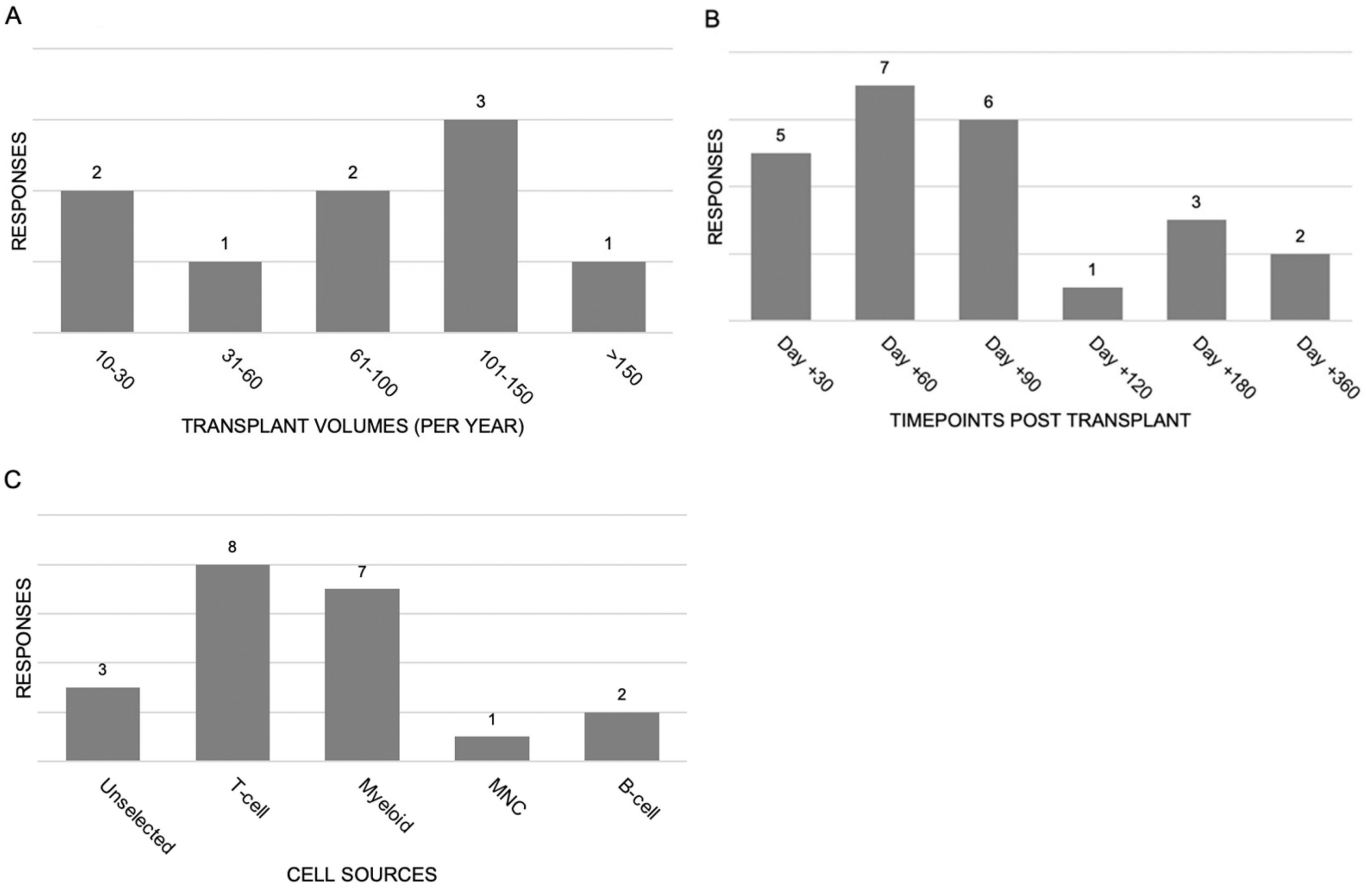
**(A)** transplant volumes completed per year by respondent centres. **(B)** Testing timepoints for chimerism post-transplant. Common testing time points include 1 month, 2 months, 3 months and 1 year post-transplant. **(C)** Cellular sources for chimerism testing. Samples were obtained from both peripheral and bone marrow source. Unselected did not separate cellular lineages. T-cell was detected by CD3+, myeloid by CD33+, CD66b+ or CD15+, B-cell by CD19+. Mononuclear cells were unselected.

Responses were received from 9 of the surveyed programs (56%). Nine of twelve (75%) programs treating adult patient populations responded, while no responses were received from pediatric or combined programs. The volume of transplants completed annually at the respondent programs ranged widely (Figure 1A). Most programs were mid (61–100) to large volume (>100), which represented 56% of responses; one program reported over 150 transplants per year.

### 3.2 Testing methods

The most frequent testing method was STR-PCR (77.7%), with one centre using both XY-FISH and STR-PCR. Variable number tandem repeat-PCR (VNTR-PCR) was used at one centre. One centre utilized real time quantitative PCR. At the time of survey, next generation sequencing (NGS) testing was standard at one centre. All centres used peripheral blood for chimerism testing, with 60% reporting additional use of bone marrow samples. Turnaround time was between one to two weeks in 60% of centres with only one centre receiving results in less than one week. The remainder reported receiving testing results between two to three weeks (20%).

### 3.3 Clinical indications and time points

All centres reported performing routine chimerism testing post allogeneic HSCT, however, three centres only tested chimerism in transplants performed with NMA or reduced intensity conditioning (RIC) regimens. As shown in Figure 1B, the most common timepoints for chimerism assessment within the first-year post-transplant were day +60 (*n* = 7), day +90 (*n* = 6), and day +30 (*n* = 5). Two centres routinely tested chimerism at one-year post-transplant while no centres reported routine testing beyond this time point. At one centre, transplants performed with NMA regimens were routinely monitored with monthly testing until full donor chimerism was achieved while another centre reported continued testing post day +120 if donor chimerism remained below 95%.

In addition to chimerism testing, routine post-transplant disease assessment with bone marrow biopsy and aspirate was performed in most centres by day +100 (44%) with two centres testing regularly at days +30 and +100 and one centre testing regularly at day +30. The remaining two centres reported only completing bone marrow biopsy and aspirates for disease assessment on an individualized basis determined by clinical indications, rather than standardized uniform testing of all patients.

### 3.4 Lineage separation

Figure 1C shows the cumulative responses for lineages tested amongst transplant centres. Almost all centres tested separate myeloid (CD33+, CD66ab+ or CD15+) populations (78%) as well as T-cell (CD3+) subsets (89%) for chimerism evaluation. Three centres reported lineage agnostic testing (whole blood) in addition to lineage specific testing, including one which tested mononuclear cells after Ficoll separation. B-Cells (CD19+) were selected specifically in two centres in patients transplanted for B-cell malignancies. Notably, all centres reported testing at least two cellular sources.

### 3.5 Clinical interventions

All but one centre (89%) indicated that chimerism results impacted the decision for interventions to prevent relapse of disease post HSCT. These included early tapering of immunosuppression (67%), DLI (78%), or initiation of post-transplant therapies such as hypomethylating agents (33%). One centre, which tested chimerism only at day +90, relied on testing results to also inform possible CD34+ cell boost rather than to monitor for engraftment. In contrast, one centre indicated that chimerism testing was only performed for engraftment monitoring and did not base clinical decisions on chimerism results.

## 4 Discussion

Chimerism testing remains an integral part of the HSCT process with many implications for post-transplant disease monitoring and therapy (6, 13, 14). While there have been recommendations made by the National Marrow Donor Program (NMDP) and the previous International Bone Marrow Transplant Registry (now Centre for International Blood and Marrow Transplant Research) over two decades ago, this survey demonstrates the heterogeneity in numerous aspects of chimerism testing across transplant centres nationally (4). In particular, the appropriate time points for chimerism testing, optimal testing methods to use, and the treatment decisions made based on testing results showed notable variation amongst the centres that were surveyed. While the survey collected data from just approximately half of all transplant centres across Canada, over two-thirds of the programs treating adults responded with representation from programs of all sizes. Additionally, all centres had an academic affiliation to research institutions, highlighting the collaborative nature of chimerism testing between translational and clinical practice. We therefore present our data of chimerism testing practices in Canada in the context of currently published literature to promote greater interest in establishment of common practices.

### 4.1 Testing methodology

Chimerism testing involves the identification of polymorphic regions of DNA that differ significantly between donor and recipient to evaluate the relative percentage of hematopoietic cells of either origin (11) (Table 1). These polymorphic regions vary greatly in the population and can be used to generate a specific signature that can be followed in the post-transplant setting. STR-PCR was reported to be utilized most frequently, consistent with it being recognized as the current gold standard among centres worldwide (9). STR-PCR detects short tandem repeats, or 4–6 base pair units that repeat in a given genetic segment, to define both donor and recipient lineages. These repeats are highly variable and polymorphic and using PCR techniques can be amplified to detect varying levels of donor and recipient derived DNA, thus making it possible to compare the relative contribution of each in the hematopoietic compartment (15, 16). Typical testing includes the use of 14–16 STR loci probes which almost always provides a sufficient number of informative markers, even if donor and recipient are related. However, lower limit of detection of detecting recipient DNA is 5% potentially impacting the ability to confirm true complete chimerism (11, 15, 17). One centre also reported the use of VNTR-PCR, which uses similar detection methods as STR-PCR to detect regions of 14 to up to 500 base pairs, though less commonly used due the relatively smaller number of alleles that can be interrogated (11). In contrast, qPCR can detect recipient DNA at a sensitivity much better than STR-PCR, in the range of 0.01% to 0.1%, though suffers in its ability to detect informative regions between donor and recipient consistently. qPCR for chimerism testing uses single nucleotide polymorphisms or small insertion/deletion polymorphisms (indel) to detect variation between genetic material in a sample and, through the use of highly sensitive real time qPCR techniques, can detect lower proportions of recipient DNA (18–20). These single nucleotide polymorphisms are similarly highly variable in the population though typically only are represented by two alleles and require testing of more than 30 markers to accurately detect the origin of either donor or recipient DNA with commercial testing kits. One study reported a negative predictive value of ∼99% with qPCR methods, as well as the ability to accurately predict relapse at a median of 38 days prior to clinical detection, largely owing to faster turnaround times compared to traditional methods (21). Notably, only one centre in our survey reported using qPCR methods.

**Table 1:** Donor/recipient chimerism detection methods.

Lab Technique	Marker	Sensitivity	Informativity
PCR (16, 17)	STR, VNTR	1%–5%	∼100%
qPCR (19, 20)	SNP, indels	0.1%–1%	95%–100%
FISH (9, 11)	X/Y chromosome	<5%	100%
NGS (3, 11, 22)	SNP, indels	0.01%–0.1%	∼100%

a Summary of commonly adopted DNA detection methods to assess chimerism. Sensitivity represents detection limit of recipient DNA by testing method. Informativity represents collective ability of markers to distinguish between donor and recipient DNA.

There was one centre that utilized FISH X-Y chromosome testing to evaluate chimerism, though this strategy can only be performed in sex mismatched transplantation and is more labour intensive compared to molecular methodologies. While it remains a viable technique to detect the presence of donor cells of a different sex, this requirement renders it less useful than techniques that are sex chromosome agnostic.

NGS chimerism panels detect common single nucleotide variants (SNVs) differences between donor and recipient DNA (3). Currently, there is only one centre in Canada that reported the use of NGS to evaluate donor chimerism as their standard, with ongoing expansion of this technique into other areas of hematology suggesting its increased application in post-transplant monitoring. Given the improvement in ability to detect recipient chimerism of <0.01%, NGS testing can detect very slight reduction in donor chimerism below 100%. Comparatively, larger decreases in donor chimerism using STR detection are required to assess increasing recipient DNA where traditional thresholds are 1%–5%. NGS methods therefore have the potential for earlier detection of impending disease relapse or recurrence. Capital investment into NGS infrastructure and more rapid analysis pipelines are barriers to uptake and utilization. High-resolution human leukocyte antigen typing and mutational/measurable residual disease (MRD) testing also utilizes NGS techniques and the incorporation of chimerism testing will create opportunities for increased utilization amongst transplant centres.

While the largest proportion (*n* = 7) of testing centres continue to use STR-PCR as the preferred method of chimerism analysis, novel techniques of interrogating the genetic origin of cell lineages will improve the sensitivity of detection for lower levels of recipient hematopoietic cells. Additionally, turnaround time remains a significant issue with chimerism testing, with some studies noting relapse in as little as 30 days post sample collection which may leave limited time between receipt of results and overt disease relapse (21, 22). Novel testing techniques may provide quicker results to more promptly and accurately evaluate falling donor chimerism allowing for intervention prior to overt morphologic disease recurrence. This could be of particular interest for several of the centres that reported prolonged turnaround times of greater than 2 weeks. Canadian transplant centre laboratories are accredited according to American Society for Histocompatibility and Immunogenetics standards and if consensus is reached between centres on which techniques to use, this will prove useful for ongoing research into the use of chimerism in disease relapse monitoring.

### 4.2 Engraftment and relapse monitoring

Donor stem cell engraftment after HSCT is paramount to the successful treatment of hematologic disease without ongoing cytopenia and has historically been the primary reason for chimerism testing. Additionally, monitoring of donor engraftment aids in the detection of impending disease relapse and can allow for clinical intervention. The American Society for Transplant and Cellular Therapy (ASTCT) has published panel guidelines aimed to standardize definitions of post-transplant engraftment where full donor chimerism is defined as 95% donor derived lymphoid and myeloid cells (23) likely in keeping with the sensitivity of the most common testing methods (e.g., STR-PCR). Despite these published guidelines, specific time points for chimerism testing such as day +30 or at time of blood cell count recovery to meet the formal definition of engraftment remains heterogenous as highlighted by our survey responses.

Early chimerism monitoring is largely completed to ensure adequate engraftment and graft hematopoietic function, however, important prognostic information can be obtained as well. Multiple studies have indicated that donor chimerism <90% at day +30 is associated with higher rates of relapse, decreased progression free survival and poorer OS (6, 13, 22, 24, 25). A retrospective study showed that mixed whole blood chimerism at day +30 was significantly associated with incidence of relapse, relapse free survival (RFS) and overall survival (OS) with regression analysis showing an almost 10% improvement in relapse risk with every 1% increase in chimerism (25). While day +30 has been demonstrated to be prognostically relevant, the chimerism dynamic overtime can provide even greater prognostic information, supporting the recommendations for multiple measurements post-HSCT rather than at one single time-point (6, 21). Rationale for the selected measurement timepoints at each center was not elucidated in the survey and therefore it is not known why more than half of the centers do not routinely measure day +30 chimerism.

In contrast, two-thirds of the respondent transplant centres in Canada reported testing at day +90 which is in keeping with what has been reported internationally (9, 23). Chimerism engraftment dynamics from day +30 to +90 can provide additional information on the risk of relapse, further supporting the rationale for testing at this time point (6, 21, 26). Several factors, such as the conditioning intensity, use of T-cell depletion and graft cell dose (both CD34+ and CD3+), have been identified as factors that can impact post-transplant chimerism (27, 28). In a study with *in-vivo* T-cell depleted grafts utilizing alemtuzumab, the proportion of bone marrow complete chimerism in unselected subsets at 3 months was 32% in NMA conditioned transplants compared to 71% utilizing myeloablative conditioning (28). This suggests that, after initial suppression of recipient alloimmunity against donor cells by the conditioning regimen, there is a repopulation of recipient derived hematopoiesis that results in incomplete donor chimerism by day +90. Interestingly, the use of DLI in the previously mentioned study was effective in establishing bone marrow complete chimerism in the setting of previous mixed chimerism, again indicating the importance of graft alloreactivity against recipient-derived hematopoietic stem cells to obtain, and maintain, complete chimerism.

A similar number of centres that tested at day +90 reported testing at day +60. After controlling for the day +30 chimerism (mixed/complete) result, the development of mixed whole blood or T-cell chimerism at day +60 has been shown to have a significant negative impact on RFS (6). This result was similarly demonstrated with mixed whole blood chimerism at day +90, but not day +90T-cell chimerism. Measurement at this time point allows for evaluation of ongoing donor hematopoiesis in the presence of cytopenias and following adjustments in immunosuppressants and therapeutic targets (29). Studies have shown day +60 platelet count to be prognostic for transplant related outcomes, but the association of cytopenias with mixed chimerism has not been robustly evaluated (30, 31). Similarly, in pediatrics, day +90 mixed chimerism, specifically in the T-cell lineage, has been shown to be common and not associated with graft failure or relapse when stable. However, development of mixed donor B-cell chimerism (not T-cell or myeloid) at or after day +90 has been shown to be associated with an increased risk of relapse in patients with hematologic malignancies. While age has not commonly been associated as a predictor of mixed chimerism in adults, patients younger than 3 years of age have been shown to have significantly lower donor chimerism in whole blood and myeloid lineages at day +90, potentially attributable to differences in pharmacokinetics of drugs between infants and older children/young adults (32).

Testing beyond day +90 was not consistently completed with two centres testing at a single time point each: one at day +120 and one at day +180, while two centers tested at both day +180 and +360. Recent recommended consensus published in the UK suggest frequent chimerism testing at an interval of every 3 months until at least 2 years (33). This more frequent testing in non-malignant disease can be used to guide immunosuppression tapering which is frequently continued for longer than in malignant processes (33). Other indications where clinical rationale could support regular testing beyond day +180 are reduced intensity conditioning persistent mixed chimerism, ongoing immunosuppressive taper and high-risk disease with a lack of sensitive molecular MRD markers (34). The utility of testing beyond day +360 in malignant diseases is still questionable as the majority of relapses will often occur in the first-year post-HSCT and should likely be individualized rather than universal. It still remains unclear whether chimerism testing post day +180 in malignant disease can provide further clinical information compared to MRD testing with further studies needed to evaluate their complementary use (26).

The lineages tested for chimerism also varied considerably across surveyed centres, with most reporting use of a myeloid-enriched subset. Although whole blood chimerism testing allows for simpler DNA extraction, several studies have demonstrated the advantages of lineage-specific analysis (35–38). Reconstitution of various hematopoietic lineages occur at different rates in the post-transplant period which may skew test results and lead to mixed chimerism and impacting clinical decision making (32). Furthermore, *in vivo* and *ex vivo* T-cell depletion strategies, along with non-myeloablative or reduced intensity conditioning regimens, can alter both host and donor lymphoid compartments, limiting the utility of CD3+ lineage testing (32, 38, 39). In contrast, chimerism testing of myeloid progenitors using CD33+ selected subsets has been shown to more accurately predict relapse, particularly in myeloid malignancies, with complete chimerism correlating with the lowest risk of disease recurrence (40, 41). Finally, direct testing of hematopoietic stem cells with CD34+ selection has also been effective at detecting earliest mixed donor chimerism and impending morphological relapse (37), though the relatively few amount of circulating progenitor cells may impair evaluation of both donor and recipient DNA, often necessitating more invasive bone marrow sampling instead of peripheral blood testing.

### 4.3 Management of mixed chimerism

Nearly all respondents in the survey indicated that testing chimerism is relevant in guiding clinical decision-making in the post-engraftment period. Pre-emptive immunomodulation can help to restore complete donor chimerism from mixed chimerism and increase the graft-vs.-tumor effect in efforts to avert impeding disease relapse. This can be achieved through withdrawal of post-transplant immunosuppression or DLI (8, 42). Rapid tapering of immunosuppression is aimed at improving donor lymphocyte function to promote disease eradication. Kekre et al. showed improved chimerism response (defined as donor T-cell chimerism to >90%) in reduced intensity conditioning transplants after tapering of immunosuppression with approximately 30% of patients achieving complete chimerism (43). Despite the increased incidence of acute GVHD and/or chronic GVHD, this resulted in an increase in overall survival (OS) with a HR = 0.52 (95% CI 0.28–0.97, *p* = 0.039) in those responding to tapering indicating its role in the management of mixed chimerism.

Furthermore, the benefit of DLI has been shown in patients with lineage-specific molecular relapse or mixed chimerism compared to morphologic hematologic relapse, with studies showing a response of 42% in patients with MRD (assessed by mixed chimerism) compared to 16% with overt disease recurrence (44, 45). Restoration of full donor chimerism with DLI has also been shown to improve survival, with one study reporting a 1-year OS of 83% in patients who successfully converted from mixed chimerism to complete chimerism after DLI. However, this comes with the risk of new or worsening GVHD, as seen with decreasing immunosuppression, which may negatively impact patient-relevant outcomes (46).

Prospective trials have investigated hypomethylating agents, namely azacitidine, in the management of decreasing CD34+ donor chimerism (defined as <80%) as a marker for MRD and found up to 70% of patients with this MRD surrogate responding to hypomethylating agents resulting in a 12 month RFS of 46% (14, 47). Importantly, this improvement in disease control was not associated with an increase in GVHD, suggesting that enhanced donor graft function may contribute to better control of residual disease without added toxicity. More recently, there have been attempts to combine multiple therapies to address mixed chimerism by using both prophylactic DLI and hypomethylating agents for disease at higher risk of relapse. Guillaume et al. found that both DLI and prophylactic hypomethylating agents post-transplant in patients with high risk MDS/AML resulted in a cumulative incidence of relapse at 2 years of only 27.6% with a 2-year OS of ∼66% (48). Further strategies to prevent overt morphologic relapse after detection of mixed chimerism include targeted therapies (i.e., FLT-3, IDH1 inhibitors) though it remains unclear whether these strategies alone can convert patients back to full donor chimerism (49).

In contrast, the implications of mixed donor chimerism and the need for intervention in non-malignant diseases vary compared to when transplant is used for graft vs. tumor effect. While less concerning for relapse of underlying clonal disease, cytopenias or graft rejection can result in mortality from infectious and hemorrhagic complications. Several studies have shown that stable mixed chimerism, without overt graft failure, results in similar survival compared to those with complete chimerism (41, 50–52). One study showed that patients transplanted for severe aplastic anemia who had stable mixed chimerism (<5% fluctuation in recipient DNA) did not have any evidence of graft failure. Conversely, those with progressive mixed chimerism (defined as increase in <15% of recipient DNA over 3 months) had worse outcomes related to graft failure and cytopenias (53). This dynamic change in mixed chimerism progression over 3-month periods also supports increased chimerism monitoring at day +180 and day +360 in non-malignant diseases. Notably, the majority of these studies were done in the pediatric population where a greater proportion of transplants for non-malignant conditions are performed compared to adults. No respondents indicated a difference in chimerism testing practices between malignant and non-malignant diseases, though it is important to note this was not explicitly asked in the survey.

## 5 Conclusions

Chimerism testing strategies remain heterogenous amongst transplant centres in Canada, however, several key similarities exist. Chimerism analysis remains an integral part of post-HSCT monitoring with implications in graft function and disease monitoring. The majority of centres continue to use STR-PCR analysis methods, as is largely standard practice internationally as well. Multiple key time points, namely day +30, +60–70 and +90, are evaluated by most centres in Canada, allowing for dynamic assessment of chimerism levels to aid in disease prognostication and clinical intervention. Management of mixed chimerism varied across the country and represents a key area for further focused research and actionable standardization.

There are several limitations of this survey. The data is largely qualitative in nature and aimed to evaluate chimerism practices amongst Canadian transplant centres. Approximately half of the transplant centres (56%) who were invited to participate responded, which is in keeping with other qualitative survey studies (54). Respondents represented programs with a range of transplant volumes and from differing clinical settings, including a large proportion of adult transplant programs. Though there did not appear to be any strong correlation between the size of the transplant program and testing practices, outside of more frequent testing in the early post-transplant period, this survey was not powered to provide statistical inference. Another limitation is that our survey was only delivered to transplant program medical directors for completion. Despite the highly standardized operating practices and laboratory-accredited testing methods of transplant programs, there remains the possibility of intra-program variation between practitioners, particularly regarding the management of mixed chimerism. Future surveys engaging individual practitioners within a transplant program would help to formally investigate the impact that individual variability in practice may have. As well, it is important to recognize that our survey may not fully reflect practice patterns of all national centres, particularly practice patterns in pediatric transplant programs. Engaging pediatric transplant practitioners will be important for further harmonization efforts given their lack of involvement in the survey and the potential for differences that can be seen with age and treatment population.

To our knowledge, this survey provided the largest evaluation of Canadian HSCT chimerism practices to date, and characterizes common practices, while informing areas in need for establishing consensus (Table 2). Development of guidelines for chimerism testing should be based around these most common practices highlighted by the survey and serve as a framework for any standardization committee.

**Table 2:** Donor/recipient chimerism detection methods.

Chimerism testing characteristics	Canadian Common Practices^a^	International Common Practices (9)	ASTCT Consensus Recommendations (4, 24)	Proposed Consensus Recommendations
Testing Method	STR-PCR, qPCR	STR-PCR	Recommends STR-PCR or VNTR-PCR	STR-PCR, consideration of NGS if available
Testing Timepoints	Day +30, +60, and +90 post-transplant	Day +30, +90, and +180 post-transplant	Day +30, +90, +180 and 1 year post-transplant	Day +30, +60 and +90. Day+180/+360 individualized
Lineage Testing	Lineage specific; CD3+, CD33+selection	Lineage specific; CD3+, CD33+selection	Myeloid (CD33+) and T-cell (CD3+) lineage for all conditioning regimens	Lineage specific testing; CD3+, CD33+ selection
Clinical Utility of Chimerism Testing	Pre-emptive DLI and immunosuppression tapering	Hematopoietic donor cell engraftment	Consensus recommendations for donor cell engraftment, no current recommendations for additional clinical utility of chimerism testing	Engraftment monitoring, monitoring of impending disease recurrence, guidance of therapies for relapsed disease.

Recommendations lack specificity for non-malignant disease.

a Based on most common CTTC survey responses.

DLI, donor lymphocyte infusion; NGS, next generation sequencing; PCR, polymerase chain reaction; qPCR, quantitative polymerase chain reaction; STR, short tandem repeats; VNTR, variable number tandem repeats.

Developing strategies to detect disease relapse earlier and confirm adequate ongoing donor hematopoiesis for transplant recipients is essential to improve transplant outcomes. Other clinical tools such as alterations in blood counts, bone marrow morphologic abnormalities or MRD could suggest impending relapse and therefore warrant further investigation in the context of chimerism to further understand the true clinical utility of chimerism itself (26). Canada is uniquely poised to standardize and follow these testing practices given access to funding through its universal healthcare which allows for delivery of best recommendations, especially when consensus driven. Chimerism testing has been shown to be an invaluable tool to support clinicians in the management of patients post alloHSCT, with implications across the transplant continuum. Prospective trials should investigate the utility of chimerism as a predictive tool for early intervention of impending relapse and to guide use of DLI, CD34+ cell boost, or pharmacologic interventions to improve outcomes. The standardization of chimerism testing practices amongst transplant centres will also aid in collaborative research efforts to better define and interpret when and how best to detect chimerism in HSCT recipients.
