# Supplementary material for: Chimerism practices across Canadian transplant centres: review of current standards and variations
Source: Front Transplant. 2026 Jul 31;5:1879340. doi: 10.3389/frtra.2026.1879340 (PMC13473900; doi:10.3389/frtra.2026.1879340)
Supplement: Supplementary file 1 [file Datasheet1.pdf]

Canadian Chimerism Testing Practices

You are invited to take part in this study investigating post allogeneic stem cell transplant chimerism testing practices across Canadian transplant centres. The objective of this study is to understand the protocols, methodologies, and frequency of chimerism testing at allogeneic transplant centres across Canada. We also want to further understand disease monitoring practices after allogeneic stem cell transplant.

We thank you in advanced for your participation

Please provide email address

____

Do you consent to proceeding with this survey and that the answers will be used for research purposes?

- ◯ Yes
- ◯ No

Allogeneic Transplant Centre Information

Approximately how many allogeneic transplants are completed at your centre annually?

- ◯ 10-30
- ◯ 31-60
- ◯ 61-100
- ◯ 101-150
- ◯ >150

Which age demographics do you treat?

- ☐ pediatric (< 18 years of age)
- ☐ adult (>18 years of age)

(Select all that apply)

Does your centre complete donor chimerism testing post allogeneic transplant?

- ◯ Yes
- ◯ No

Transplant Centre Chimerism Practices

Does intensity of conditioning chemotherapy determine who has chimerism testing completed?

- ◯ yes - only completed with non myeloablative/reduced intensity conditioning regimens
- ◯ no - testing is completed on all allogeneic transplants

What methods are used for chimerism testing at your centre?

- ☐ Short Tandem Repeats (STR) - PCR
- ☐ Real Time qPCR
- ☐ Next Generation Sequencing (NGS)
- ☐ Digital Droplet PCR
- ☐ Variable Number Tandem Repeats (VNTR) - PCR
- ☐ XY-FISH

(Select all that apply)

What samples source(s) are used for testing chimerism at your centre?

- ◯ Bone Marrow
- ◯ Peripheral blood
- ◯ Both

Which lineages are utilized for chimerism testing at your centre?

- ☐ Unselected
- ☐ T-cell (CD3+)
- ☐ Myeloid (CD33+, CD66b or CD15+
- ☐ CD34+ cells
- ☐ Other

Please specify other	__________________________
What is the average turnaround time for chimerism testing?	◯ < 1 week<br>◯ 1-2 weeks<br>◯ 2-3 weeks<br>◯ > weeks
What is the standard timing for evaluating disease by bone marrow biopsy and aspirate after allogeneic stem cell transplant at your centre?	☐ At engraftment<br>☐ Day +30<br>☐ Day +100<br>☐ Not routinely completed, individualized based on clinical indications<br>(Select all that apply)
Do you base any interventions on chimerism testing?	☐ No<br>☐ Early tapering of immunosuppression<br>☐ Donor lymphocyte infusion (DLI)<br>☐ Pre-emptive therapy (eg. hypomethylating agent, target agent)<br>☐ Other<br>(Select all that apply)
Please specify other intervention	__________________________
Which chimerism testing timepoints are most frequently utilized in the first year post transplant?	☐ Before day +30<br>☐ Day +30<br>☐ Day +60<br>☐ Day +90<br>☐ Day +180<br>☐ Day +270<br>☐ Day +360<br>☐ Other
Specific other timepoint	__________________________
Do you complete chimerism regularly after 1 year?	◯ Yes<br>◯ No
What is the frequency of testing chimerism after 1 year?	☐ Monthly<br>☐ Every 3 months<br>☐ Every 6 months<br>☐ Annually<br>☐ Other
Please specify other timepoints tested after 1 year	__________________________
Any additional comments regarding institutional practices surrounding chimerism testing which may be relevant and not capture in the survey?	◯ Yes<br>◯ No
Please provide additional comments	__________________________
Please provide reason for not completing chimerism testing post transplant	__________________________
